# Bowel function after surgery in male children with complicated anorectal malformations

**DOI:** 10.3389/fped.2025.1621535

**Published:** 2025-06-12

**Authors:** Hanbin Zhao, Jian Cao, Jinping Hou, Yuan Shi, Yi Wang

**Affiliations:** ^1^ Department of General Surgery &Neonatal Surgery, Children’s Hospital of Chongqing Medical University, Chongqing, China; ^2^ Department of Neonatology, Children’s Hospital of Chongqing Medical University, Chongqing, China

**Keywords:** anorectal malformations, bowel function, spinal cord anomalies, operation intervals, perianal muscle

## Abstract

**Background:**

Some children may experience defecation dysfunction following surgery for anorectal malformations. This study evaluated the long-term functional outcomes and influencing factors in male children with complicated anorectal malformations (ARMs).

**Methods:**

We retrospectively analyzed the clinical data of male children with complicated ARMs who underwent staged surgeries in our hospital from 2013 to 2016. Data collected included ARM type, perianal muscle development, anorectal manometry findings, lumbosacral MRI findings and the intervals between the first and third operation. Bowel function after the operation was assessed by questionnaire (modified Rintala score). Logistic regression analysis was used to analyze the influencing factors of prognosis.

**Results:**

Fifty-eight children were included in this study, with a median age at PSARP of 6.7 months. Bowel function did not differ based on anorectal malformations subtype (*p* = 0.212). Perianal muscle development was significantly associated with bowel function (*p* = 0.023, *r*_s_ = 0.297). Fifty-one children received anorectal manometry, which showed no significant differences in anal resting pressure (ARP) among the different bowel function groups(*p* = 0.666). Rectoanal inhibitory reflex (RAIR) was present in 3/12 (25%), 10/36 (27.8%), and 1/3 (33.3%) children in the normal, good, and fair groups, respectively (*p* = 0.781). Bowel function was significantly worse in children with spinal cord anomalies than in children with normal spinal cord (OR = 4.651, *p* = 0.032). Stooling level worsened with increasing intervals between the first and third operation (OR = 3.808, *p* = 0.039).

**Conclusion:**

The incidence of spinal cord abnormalities in male children with complicated ARMs was high. Spinal cord anomalies and long intervals of the staging operation increased the risk of poor postoperative outcomes.

## Introduction

Anorectal malformations (ARMs) are a rare gastrointestinal abnormality that occurs with an incidence of approximately 1 in 5,000 live births (1). Improved surgical techniques and postoperative management have led to gradual improvements in children's bowel function. However, postoperative defecation disorders occur in most cases, including fecal incontinence and constipation, which affect quality of life and cost of care (2–4). Long-term bowel function in children with ARMs is influenced by multiple factors, including the degree of sacral development and the integrity of pelvic musculature (5). Investigating anatomical and neurological abnormalities is vital for predicting functional outcomes and planning long-term follow-up, yet few studies have focused on their effects in different sexes.

Regardless of the classification of the ARM, male and female children have their own unique pathological types. Due to the anatomical differences in the urogenital system, the distribution of pelvic floor muscles varies between male and female children with ARMs. Furthermore, some children with congenital rectovestibular fistula, which have a higher incidence, underwent single-stage surgery. As a result, female children are not suitable for inclusion in this study. The surgical techniques and prognosis differ based on the type of fistula. High and intermediate anorectal malformations require a three-stage surgical approach: colostomy, PSARP, and colostomy closure. Consequently, we describe male children with ARMs that necessitate this three-stage surgery as complicated anorectal malformations. The aim of this study was to provide information on the clinical characteristics and determine the influential factors on the functional outcomes in male children with complicated ARMs.

## Patients and methods

### Patient population

Following ethics approval by Institutional Review Board of Children's Hospital of Chongqing Medical University (#053/2014). We conducted a retrospective review of all male children who presented between October 2013 and September 2016 in our center and were diagnosed with complicated ARMs. The inclusion criteria for this study were as follows: (1) male children who were diagnosed with ARMs; (2) children who underwent staged surgeries; and (3) parents who expressed a willingness to participate in the study. The exclusion criteria included: (1) children with an interval between colostomy closure and the last follow-up that did not exceed 8 years; (2) children who voluntarily withdrew, refused to cooperate, lost contact, or died during the follow-up period.

### Preoperative workup

All children had received lumbosacral magnetic resonance imaging (MRI) before the PSARP. The diagnosis of spinal cord anomalies via MRI was conducted by experienced radiologists. Anorectal manometry was performed using the ManoScan gastrointestinal dynamics detection system after the colostomy closure. Enema was performed on the morning of the examination. Anorectal manometry was performed under sedation with the patient in the lithotomy position. First, anal resting pressure (ARP) was collected, and the balloon was gradually dilated starting from 10 ml with the manometry catheter until rectoanal inhibitory reflex (RAIR) occurred or the maximum capacity was 60 ml.

### Intraoperative examination

**Figure 1:**
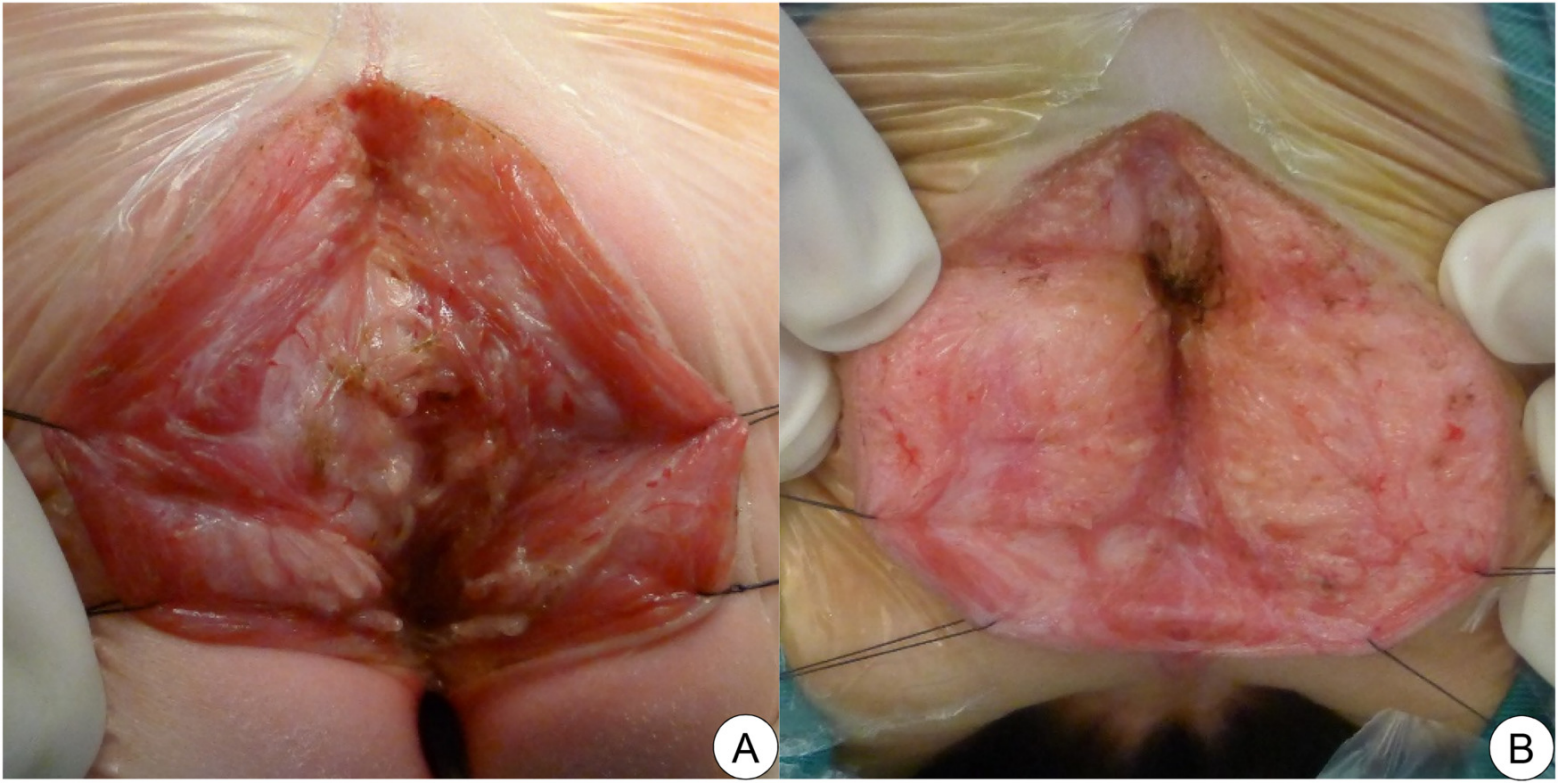
Examples of different levels of perianal muscle development in male children with complicated ARMs. **(A)** Good development of perianal muscles. **(B)** Poor development of perianal muscles.

All children underwent PSARP by the same pediatric surgeon. Images of perianal muscles (parasagittal fibers, muscle complex, levator muscles) were collected during the operation. Images were blindly evaluated by two pediatric anorectal surgeons. Good development was defined as thick muscle fibers, clear texture and less adipose tissue. Poor development was defined as slender muscle belly, blurry texture and a bulk of adipose tissue (Figure 1). Between these two assessments was defined as moderate development.

### Postoperative follow-up

We started anal dilatations 2 weeks after PSARP and encouraged the parents to continue doing the dilatations twice a day. Meanwhile, the size of the dilator was increased by one size per week (1 mm per week) until they reached the size that was adequate for the age of the children. Thereafter, the parents were supposed to start tapering the frequency of the dilatations. They were to change the routine from twice a day to once a day for a month, and then every other day for a month, twice a week for a month, once every 2 weeks for a month, and then stop the dilatations (6). All children were followed up in the anorectal clinic of our hospital and for defecation and diet management for at least 5 years. Functional outcome were evaluated through either telephone interviews or in-person interviews utilizing the modified Rintala bowel function score (4). Total scoring ranged between 0 and 17 and was secondarily classified into 4 groups: normal, 15–17; good, 10–14; fair, 6–9; and poor, 0–5.

### Statistical analysis

Statistical analysis was performed using SPSS (IBM SPSS Statistics for Windows, IBM Corp., Version 27.0, Armonk, NY). Normally distributed variables were expressed as $\bar{X}\pm S$. Skewed distributed variables were recorded as medians and ranges. Quantitative data were compared with the Mann–Whitney *U* test. Ranked data were compared with Mann–Whitney *U* test or Spearman's correlation. *P* < 0.05 was regarded as statistically significant. An ordinal logistic regression model was used to calculate odds ratios and 95% confidence intervals.

## Results

### Demographic characteristics

There were 58 children in the study group. All children underwent a three-stage surgical procedure, which included colostomy (age range, 1 day to 1 month; median age, 2 days), posterior sagittal anorectoplasty (PSARP) (age range, 5 months to 1.6 years; median age, 6.7 months) and colostomy closure (age range, 8.5 months to 2 years; median age, 11 months). Twenty-five children had associated malformations. The most frequent malformations were cardiovascular system malformations (20 children, 34.5%), including atrial septal defect in 15 children (75.0%), patent ductus arteriosus in 2 children (10.0%), ventricular septal defect in 1 patient (1.7%), and aortic valve stenosis complicated with atrial septal defect in 1 patient (1.7%), patent ductus arteriosus complicated with atrial septal defect in 1 patient (1.7%). Other malformations included unilateral agenesis of the kidney (5 children), polydactyly (2 children) and 21 trisomy syndrome (2 children; both without a fistula).

### Functional outcome results

Fourteen out of 58 children (24.1%) had a normal Rintala score, 39 (67.3%) had a good score, and 5 (8.6%) had a fair score. No patient had a poor score. No significant differences were observed based on the anatomic type of ARM according to the Krickenbeck classification (7) (*p* = 0.212) (Table 1).

**Table 1:** Univariate analysis of risk factors of bowel function after surgery.

Items	No. patients (%)	Bowel function			*P*
		Normal	Good	Fair	
ARM subtype					0.212
ARM without fistula	10 (17.2%)	3	7	0	
Rectobulbar fistula	27 (46.6%)	3	21	3	
Rectoprostatic fistula	20 (34.5%)	8	10	2	
Rectobladder neck fistula	1 (1.7%)	0	1	0	
Lumbosacral MRI					0.020
Anomalies	19 (32.8%)	3	11	5	
Normal	39 (67.2%)	11	28	0	
Perianal muscles					0.023
Poor	4 (6.9%)	0	1	3	
Moderate	47 (81.0%)	12	33	2	
Good	7 (12.1%)	2	5	0	
RAIR					0.781
Positive	14 (27.5%)	3	10	1	
Negative	37 (72.5%)	9	26	2	
Interval of three-stage operation					0.017
≤11 months	33 (56.9%)	12	19	2	
>11 months	25 (43.1%)	2	20	3	

### Lumbosacral MRI

Of the 58 children, 19 (32.8%) showed anomalies in spinal cord development, including fatty filum terminale in 9 children (15.5%), intraspinal lipoma in 4 children (6.9%), intraspinal cystic lesions complicated with fatty filum terminale in 1 patient (1.7%), and syringomyelia complicated with intraspinal lipoma in 1 patient (1.7%), low position of conus medullaris in 4 children (6.9%). Negative MRI was demonstrated in 39 out of 58 children (67.2%), and the functional outcome significantly differed between the children with normal and those with abnormal spinal cord development (*p* = 0.020).

### Development of perianal muscles

**Figure 2:**
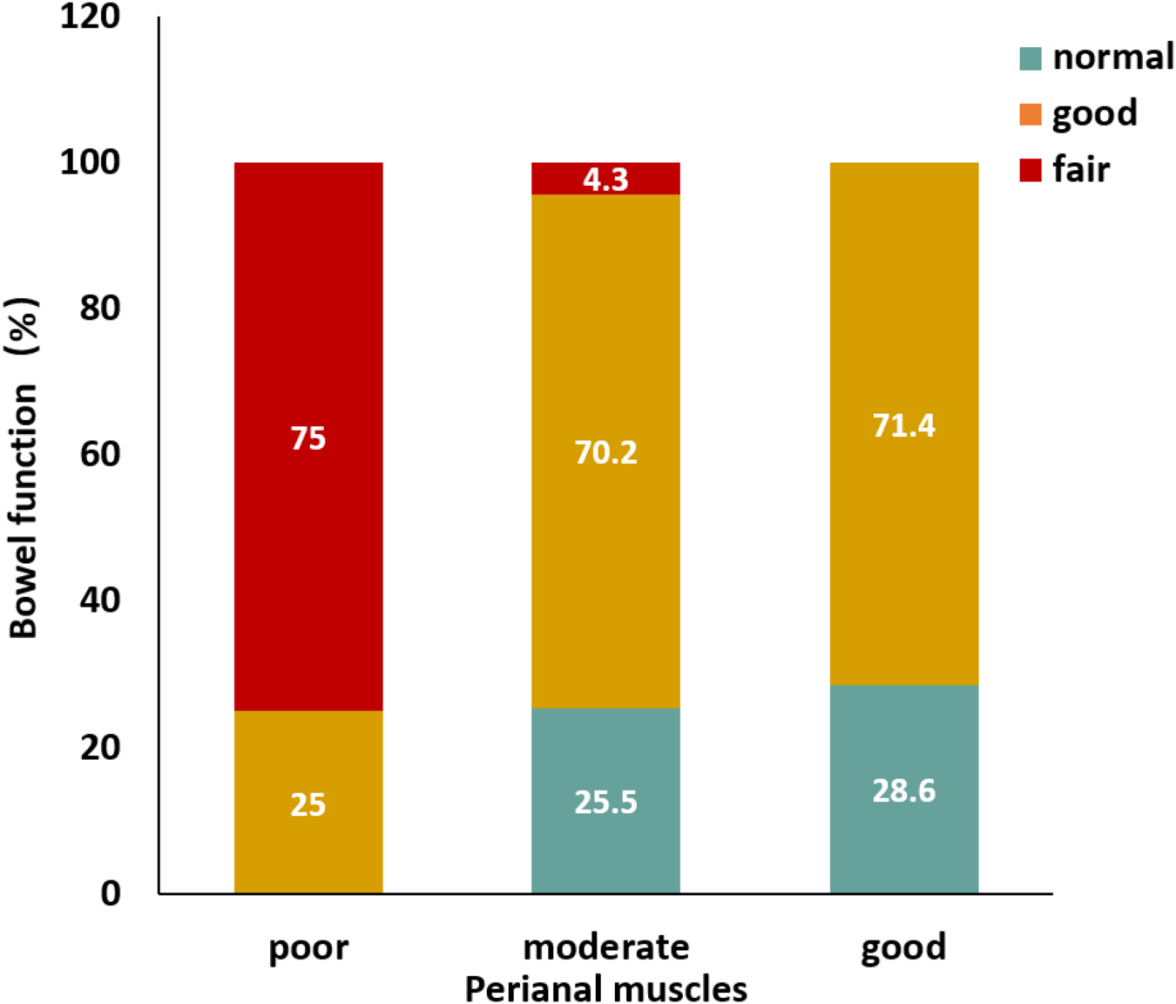
Distribution of bowel function under varying conditions of perianal muscle development.

According to the images collected during surgery, 7 children (12.1%) had good development, 47 (81.0%) had moderate development, and 4 (6.9%) had poor development (Figure 2). A significant correlation was noted between the functional outcome and development of perianal muscles (*p* = 0.023, *r*_s_ = 0.297).

### Anorectal manometry

**Figure 3:**
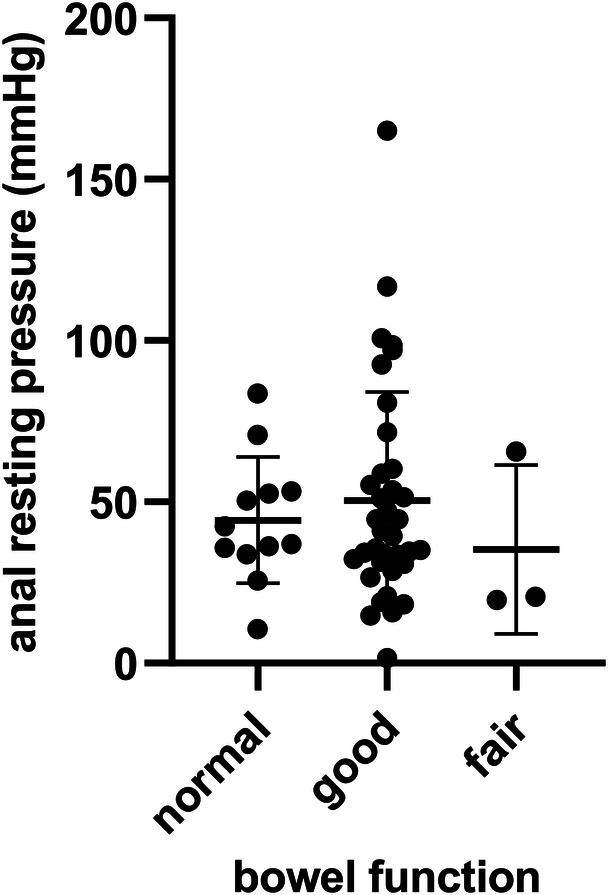
Anal resting pressure in children with different bowel function.

Fifty-one children underwent anorectal manometry. The presence of RAIR was demonstrated in 3 of 12 children (25.0%) in the normal group, in 10 of 36 (27.8%) cases in the good group and in 1 of 3 (33.3%) cases in the fair group. However, there were no significant differences in the results of the positive rate of RAIR (*p* = 0.781) among the 3 groups. The mean ARP for each group was as follows: normal, 44.31 ± 5.66 mmHg; good, 50.44 ± 5.61 mmHg; fair, 35.23 ± 15.14 mmHg (Figure 3). There were no significant differences in the median ARP among the 3 groups (*p* = 0.666).

### The interval between the first and third operation

The interval between the first and third operation ranged from 4 months to 2 year (mean of 11 months), and the interval was ≤ 11 months in 33 children (56.9%) and >11 months in 25 children (43.1%). The functional outcome differed significantly between the 2 groups (*p* = 0.017).

### Multivariate analysis

We used an ordinal logistic regression based on the univariate analysis, and the results showed that anomalies in spinal cord development and an interval between the first and third operation that was >11 months were the main risk factors influencing functional outcomes of male children with complicated ARMs (Table 2).

**Table 2:** Multivariate analysis of risk factors of poor bowel function after surgery.

Variable	B	SE	Odds ratio (95% CI)	*p*
Spinal cord anomalies	1.537	0.715	4.651 (1.146–18.878)	0.032
Interval of three-stage operation	1.337	0.647	3.808 (1.071–13.518)	0.039

## Discussion

Anal reconstruction surgery is not only to establish a defecation channel, but more importantly to improve the child's defecation function. Previous studies on bowel function of children with ARMs were based on the Wingspread or Krickenbeck classification, but they rarely described function across sexes (7, 8). Regardless of which classification of ARMs, male and female children have their own unique pathological types, and the anatomical structure of pelvic floor differs. We analyzed the influencing factors of bowel function only in male children with complicated ARMs. All children underwent 3 surgical procedures, and the second operation was performed by the same doctor. Thus, the results are high in reliability.

Clinical evaluation revealed that 14 of the 58 children (24.1%) had a normal Rintala score, and no patient had a poor score. This is much better than the report of Rintala (normal, 34.8%; poor, 8.7%) (4). These differences may have been caused by improvements in surgical skill and the enhancement of postoperative management. The differences in bowel function between the different ARM groups was not statistically significant and contradicts previous research conclusions, but the current study contained all male children with complicated ARMs. The comparison range is small, and the results may be more realistic.

The embryonic association of ARMs and anomalies in the spinal cord has been proven by Suppiej et al. (9). Previous studies have shown that abnormal development of the lumbosacral spinal cord may cause dysfunction of the anus and pelvic floor muscles in the embryo, thus promoting the occurrence of ARMs (10). The association of anomalies in spinal cord development and ARMs has been described with a prevalence between 26.8% and 51.0% (11–13), and the rate of anomalies in the spinal cord was increased in those with increasing complexity of ARMs (14). In our study, spinal cord anomalies were found in 19 (32.8%) of the 58 children. The most frequently occurring anomaly was found to be fatty filum terminale (15.5%). This indicated the high incidence of spinal cord anomalies in male children with complicated ARMs. Spinal cord anomalies can lead to traction and compression on the primary defecation center that might result in defecation dysfunction (15).

However, the effect of spinal cord anomalies on functional prognoses had not been well investigated in the literature. Some authors have reported that spinal anomalies were not associated with a poor functional prognosis (16, 17). Our present results echo those of Wong et al. (18) and Kyrklund et al. (19), who reported that children with spinal cord anomalies had a worse functional prognosis than children without spinal cord anomalies. It has been reported that some children may experience worsening of spinal cord lesions, as well as abnormal sensory and motor functions in the lower limbs during follow-up assessments. Surgical intervention for symptomatic spinal cord abnormalities has the potential to offer relief (16, 20). Currently, there is controversy about prophylactic untethering surgery in children with ARM associated with asymptomatic spinal cord anomalies; there is evidence that prophylactic surgery has only a limited influence on functional outcome, but it will have a certain risk, such as intracranial infection, cerebrospinal fluid leakage, or aggravated neural deficiencies (19, 21). Therefore, we recommend that early routine MRI examination and long-term follow-up and bowel management after the operation is essential for male children with complicated ARMs. The operative opportunity for untethering surgery for asymptomatic spinal cord anomalies requires a further prospective randomized clinical trial.

Defecation control is achieved through the contraction and relaxation of perianal muscles. Children with ARMs not only have anorectal agenesis but also have poorer perianal muscle development than normal children (22). PSARP involves complete exposure of the perianal muscles by means of a median sagittal incision that runs from the sacrum to the anal dimple (23). Our study demonstrates that bowel function after surgery in male children with complicated ARMs was associated with perianal muscle development based on a univariate analysis. However, in the multivariable analysis that included the spinal cord anomalies and operative interval, perianal muscles were not independently associated with bowel function. This indicated that the influence of perianal muscles on bowel function may be overshadowed by the spinal cord anomalies or operative interval. Therefore, to predict bowel function after surgery, it is quite essential to evaluate the perianal muscle development of male children with complicated ARMs.

Anorectal manometry is an approach to assessing the compliance, defecation reflex and sensory mechanisms of the anorectal system. Previous investigations have revealed that children with lower ARP and impaired RAIR have worse functional outcomes (24, 25). Based on this, the bowel management plan could be changed, and electric stimulation or biofeedback training would be added to improve the bowel function of ARM children after operation. In our study, the ARP and rate of RAIR were not different among the children with normal, good and fair Rintala scores. To minimize the likelihood of non-cooperation among some children, anorectal manometry was conducted under sedation, whereas the Rintala assessment was performed in an awake state. However, postoperative anorectal manometry is still the most visual index to evaluate the pelvic floor muscles, which can provide more realistic information about future bowel function and might help in making the treatment strategies. We will continue to follow up these children and conduct further examinations when they are able to cooperate with the anorectal manometry to eliminate potential errors.

In 1982, de Vries and Pena (1) introduced the posterior sagittal anorectoplasty (PSARP) for ARM children. They also advocated to perform a colostomy before PSARP to prevent wound infection, which prevented harm to the anal sphincter complex and perianal nerves after reconstruction of the anus. Since then, the three-stage surgical procedure, which included colostomy, has been the mainstay of therapy for intermediate or high ARMs. However, the choice of one-stage anorectoplasty or the conventional multistage approach has always been a hot topic in the treatment of intermediate or high ARMs (26). Several authors have shown that the advantages of one-stage anorectoplasty over three-stage surgical procedures are low cost, shorter period of treatment and no colostomy-related complications (27). Colostomy surgery in children with ARMs may impact the neural development of the distal colon (28). Moore also described the presence of the brain-defecation reflexes (29–31). By restoring gastrointestinal continuity early, bowel function is trained early, and synapses and neuronal frameworks may be established, which may result in better functional outcomes. However, Peña believed that colostomy-related complications can largely be avoided by meticulous technique and stoma management (32). The extant literature describes the rising risk of wound infection and wound dehiscence without protective colostomy that might result in damage to the sphincter and worse function (26, 33, 34).

In our series, all children underwent three-stage surgical procedures, which included colostomy, posterior sagittal anorectoplasty and colostomy closure, and no patient presented with perianal infection, anal stenosis, recurrence of fistula, or severe colostomy-related complications. In our children, the interval between the first operation and the third operation is the time period during which gastrointestinal continuity was interrupted, and this interval was also the time period during which the brain-defecation reflex was not being trained. Statistical analyses show that children with intervals greater than 11 months had a worse functional prognosis than children with intervals less than or equal to 11 months. This result may have been caused by the long period of not training the brain-defecation reflex. Therefore, the three-stage surgical procedures for male children with complicated ARMs are still the treatment strategy to ensure fewer complications and better functional prognosis. At the same time, under the premise of ensuring safety, the interval between the first operation and the third operation should be shortened as much as possible, thus improving the prognosis.

This study excluded the influence of gender on defecation function, allowing for a clearer understanding of the factors affecting defecation in male children with complicated ARMs. The findings provide a foundation for optimizing the timing of surgical interventions and enhancing postoperative functional management for these children. However, the sample size of this study is relatively small. We will continue to follow up and increase the sample size to enhance the accuracy and reliability of this study.

## Conclusions

In male children with complicated ARMs, the three-stage surgical procedures are still the treatment strategy to ensure fewer complications and better functional prognosis. Spinal cord anomalies and long intervals between the first and third operations are associated with poor outcomes. Comprehensive assessments during the perioperative period, accurate surgical manipulation, short interval between the first and third operation, long-term follow-up and bowel management after the operation are essential for children with ARMs.

## Data Availability

The original contributions presented in the study are included in the article/Supplementary Material, further inquiries can be directed to the corresponding authors.

## References

[1] deVries PA Peña A. Posterior sagittal anorectoplasty. J Pediatr Surg. (1982) 17(5):638–43. 10.1016/s0022-3468(82)80126-7 7175658

[2] Davies MC Creighton SM Wilcox DT. Long-term outcomes of anorectal malformations. Pediatr Surg Int. (2004) 20(8):567–72. 10.1007/s00383-004-1231-6 15309468

[3] Levitt MA Peña A. Outcomes from the correction of anorectal malformations. Curr Opin Pediatr. (2005) 17(3):394–401. 10.1097/01.mop.0000163665.36798.ac 15891433

[4] Rintala RJ Lindahl H. Is normal bowel function possible after repair of intermediate and high anorectal malformations? J Pediatr Surg. (1995) 30(3):491–4. 10.1016/0022-3468(95)90064-0 7760250

[5] Li L Ren X Xiao H Wang C Xu H Ming A Normal anorectal musculatures and changes in anorectal malformation. Pediatr Surg Int. (2020) 36(1):103–11. 10.1007/s00383-019-04583-1 31586234

[6] Peña A Bischoff A. Surgical Treatment of Colorectal Problems in Children. Cham: Springer (2015).

[7] Holschneider A Hutson J Peña A Beket E Chatterjee S Coran A Preliminary report on the international conference for the development of standards for the treatment of anorectal malformations. J Pediatr Surg. (2005) 40(10):1521–6. 10.1016/j.jpedsurg.2005.08.002 16226976

[8] Stephens FD Smith ED Paoul NW. Anorectal malformations in children: update 1988. Birth Defects Orig Artic Ser. (1988) 24(4):1–604. 3228578

[9] Suppiej A Dal Zotto L Cappellari A Traverso A Castagnetti M Drigo P Tethered cord in patients with anorectal malformation: preliminary results. Pediatr Surg Int. (2009) 25(10):851–5. 10.1007/s00383-009-2435-6 19680666

[10] Yang Z Geng Y Yao Z Jia H Bai Y Wang W. Spatiotemporal expression of Bcl-2/Bax and neural cell apoptosis in the developing lumbosacral spinal cord of rat fetuses with anorectal malformations. Neurochem Res. (2017) 42(11):3160–9. 10.1007/s11064-017-2354-1 28712050

[11] Nievelstein RA Vos A Valk J Vermeij-Keers C. Magnetic resonance imaging in children with anorectal malformations: embryologic implications. J Pediatr Surg. (2002) 37(8):1138–45. 10.1053/jpsu.2002.34459 12149689

[12] Golonka NR Haga LJ Keating RP Eichelberger MR Gilbert JC Hartman GE Routine MRI evaluation of low imperforate anus reveals unexpected high incidence of tethered spinal cord. J Pediatr Surg. (2002) 37(7):966–9; discussion-9. 10.1053/jpsu.2002.33817 12077750

[13] Samuk I Bischoff A Freud E Pena A. Tethered cord in children with anorectal malformations with emphasis on rectobladder neck fistula. Pediatr Surg Int. (2019) 35(2):221–6. 10.1007/s00383-018-4399-x 30413919

[14] Goulin J La Barbera G Delmonte A Bonnot E Berteloot L Lozach C Revisiting anatomy of anorectal malformations with a symbolic AI segmentation method applied on diffusion MRI: the lumbosacral Plexus development and microarchitecture is different in high and low types. J Imaging Inform Med. (2025). 10.1007/s10278-024-01378-2 40251432 PMC12920947

[15] Devroede G Lamarche J. Functional importance of extrinsic parasympathetic innervation to the distal colon and rectum in man. Gastroenterology. (1974) 66(2):273–80. 4810918

[16] Totonelli G Morini F Catania VD Schingo PM Mosiello G Palma P Anorectal malformations associated spinal cord anomalies. Pediatr Surg Int. (2016) 32(8):729–35. 10.1007/s00383-016-3914-1 27372296

[17] Minneci PC Kabre RS Mak GZ Halleran DR Cooper JN Afrazi A Can fecal continence be predicted in patients born with anorectal malformations? J Pediatr Surg. (2019) 54(6):1159–63. 10.1016/j.jpedsurg.2019.02.035 30898398

[18] Wong CWY Koga H Sugita K Kato D Mutanen A Chung PHY Functional outcome in patients with anorectal malformation with recto-prostatic or recto-bulbar urethral Fistula and comparison between different surgical approaches: a multi-center study. J Pediatr Surg. (2025) 60(2):161652. 10.1016/j.jpedsurg.2024.07.037 39181779

[19] Kyrklund K Pakarinen MP Taskinen S Kivisaari R Rintala RJ. Spinal cord anomalies in patients with anorectal malformations without severe sacral abnormalities or meningomyelocele: outcomes after expectant, conservative management. J Neurosurg Spine. (2016) 25(6):782–9. 10.3171/2016.4.Spine1641 27448173

[20] Esposito G Totonelli G Morini F Contini G Palma P Mosiello G Predictive value of spinal bone anomalies for spinal cord abnormalities in patients with anorectal malformations. J Pediatr Surg. (2021) 56(10):1803–10. 10.1016/j.jpedsurg.2021.05.011 34167803

[21] Inoue M Uchida K Otake K Nagano Y Shimura T Hashimoto K Long-term functional outcome after untethering surgery for a tethered spinal cord in patients with anorectal malformations. Pediatr Surg Int. (2017) 33(9):995–9. 10.1007/s00383-017-4127-y 28779274

[22] Liang Q Lu C Liu P Yang M Tang W Jiang W. Correlation between congenital pelvic floor muscle development assessed by magnetic resonance imaging and postoperative defecation. Pediatr Surg Int. (2024) 40(1):104. 10.1007/s00383-024-05691-3 38600320

[23] Pena A Devries PA. Posterior sagittal anorectoplasty: important technical considerations and new applications. J Pediatr Surg. (1982) 17(6):796–811. 10.1016/s0022-3468(82)80448-x 6761417

[24] Kyrklund K Pakarinen MP Rintala RJ. Manometric findings in relation to functional outcomes in different types of anorectal malformations. J Pediatr Surg. (2017) 52(4):563–8. 10.1016/j.jpedsurg.2016.08.025 27624562

[25] Kumar S Al Ramadan S Gupta V Helmy S Debnath P Alkholy A. Use of anorectal manometry for evaluation of postoperative results of patients with anorectal malformation: a study from Kuwait. J Pediatr Surg. (2010) 45(9):1843–8. 10.1016/j.jpedsurg.2010.04.012 20850630

[26] Lauriti G Di Renzo D Lelli Chiesa P Zani A Pierro A. One-stage repair of anorectal malformations in females with vestibular fistula: a systematic review and meta-analysis. Pediatr Surg Int. (2019) 35(1):77–85. 10.1007/s00383-018-4378-2 30377757

[27] Xiao H Chen L Ren XH Huang R Diao M Li L. One-stage laparoscopic-assisted anorectoplasty for neonates with anorectal malformation and recto-prostatic or recto-bulbar fistula according to the krickenbeck classification. J Laparoendosc Adv Surg Tech A. (2018) 28(8):1029–34. 10.1089/lap.2017.0690 29741982

[28] Bamoria P Ratan SK Panda SS Neogi S Mandal S Kumar C Interstitial cells of cajal and ganglion cell distribution in sigmoid stomal limbs and distal rectum after stoma formation in male anorectal malformation patients undergoing staged repair. J Indian Assoc Pediatr Surg. (2025) 30(1):22–7. 10.4103/jiaps.jiaps_155_24 39968250 PMC11832098

[29] Moore TC. Advantages of performing the sagittal anoplasty operation for imperforate anus at birth. J Pediatr Surg. (1990) 25:276–7. 10.1016/0022-3468(90)90440-k 2303998

[30] Nicoll RA Malenka RC. Contrasting properties of two forms of long-term potentiation in the hippocampus. Nature. (1995) 377:115–8. 10.1038/377115a0 7675078

[31] Albanese CT Jennings RW Lopoo JB Bratton BJ Harrison MR. One-stage correction of high imperforate anus in the male neonate. J Pediatr Surg. (1999) 34(5):834–6. 10.1016/s0022-3468(99)90382-2 10359190

[32] Pena A Migotto-Krieger M Levitt MA. Colostomy in anorectal malformations: a procedure with serious but preventable complications. J Pediatr Surg. (2006) 41(4):748–56; discussion-56. 10.1016/j.jpedsurg.2005.12.021 16567188

[33] Hernández Pérez A Deltell Collomer P Abril Sánchez C Encinas Goenechea A Gonzálvez Piñera J Dore Reyes M Analysis of postoperative complications in patients undergoing anorectal malformation surgery: are there any predisposing factors? Cir Pediatr. (2025) 38(1):19–23. 10.54847/cp.2025.01.11 39812600

[34] Kurdi M Moukhtar A Elkholy M Alwassia H Bamehriz M Khirallah MG. Delayed vs. early enteral feeding after repair of congenital recto-vestibular fistula: the effect on perineal wound healing. Front Pediatr. (2022) 10:994249. 10.3389/fped.2022.994249 36683784 PMC9846780

